# Comparison of structural progression between ciliopathy and non-ciliopathy associated with autosomal recessive retinitis pigmentosa

**DOI:** 10.1186/s13023-019-1163-9

**Published:** 2019-08-01

**Authors:** Vitor K. L. Takahashi, Christine L. Xu, Júlia T. Takiuti, Mary Ben L. Apatoff, Jimmy K. Duong, Vinit B. Mahajan, Stephen H. Tsang

**Affiliations:** 1 Department of Ophthalmology, Columbia University, New York, NY USA; 2 Jonas Children’s Vision Care, and the Bernard & Shirlee Brown Glaucoma Laboratory, Departments of Ophthalmology, Pathology & Cell Biology, Columbia Stem Cell Initiative, Institute of Human Nutrition, Columbia University, New York, NY USA; 3 Department of Ophthalmology, Federal University of São Paulo, São Paulo, Brazil; 4 Division of Ophthalmology, University of São Paulo Medical School, São Paulo, Brazil; 5 Department of Biostatistics, Columbia University, New York, NY USA; 6 Byers Eye Institute, Omics Laboratory, Department of Ophthalmology, Stanford University School of Medicine, Palo Alto, CA USA; 7 Veterans Affairs Palo Alto Health Care System, Palo Alto, CA USA; 8 Department of Pathology & Cell Biology, Stem Cell Initiative (CSCI), Institute of Human Nutrition, College of Physicians and Surgeons, Columbia University, New York, NY USA; 9 Harkness Eye Institute, Columbia University Medical Center, 635 West 165th Street, Box 212, New York, NY 10032 USA

**Keywords:** Retinitis pigmentosa, Ciliopathy, Autosomal recessive, Disease progression

## Abstract

**Background:**

To evaluate and compare the progression of ciliopathy and non-ciliopathy autosomal recessive Retinitis Pigmentosa patients (arRP) by measuring the constriction of hyperautofluorescent rings in fundus autofluorescence (FAF) images and the progressive shortening of the ellipsoid zone line width obtained by spectral-domain optical coherence tomography (SD-OCT).

**Results:**

For the ciliopathy group, the estimated mean shortening of the ellipsoid zone line was 259 μm per year and the ring area decreased at a rate of 2.46 mm^2^ per year. For the non-ciliopathy group, the estimated mean shortening of the ellipsoid zone line was 84 μm per year and the ring area decreased at a rate of 0.7 mm^2^ per year.

**Conclusions:**

Our study was able to quantify and compare the loss of EZ line width and short-wavelength autofluorescence (SW-AF) ring constriction progression over time for ciliopathy and non-ciliopathy arRP genes. These results may serve as a basis for modeling RP disease progression, and furthermore, they could potentially be used as endpoints in clinical trials seeking to promote cone and rod survival in RP patients.

## Background

Retinitis Pigmentosa (RP), an inherited retinal disorder, causes progressive photoreceptor cell death, resulting in permanent vision loss. Individuals with RP usually present with night blindness, then loss of daytime peripheral vision, and eventual extreme visual impairment or blindness. Some cases rapidly progress over two decades while some have slow progression, never resulting in actual blindness. The prevalence of RP is approximately 1 in 3500–4000 [1]. The disease can be inherited in an autosomal recessive (50–60%), autosomal dominant (30–40%) or X-linked (5–15%) manner [2]. Thus far, at least 64 genes (RetNet; https://sph.uth.edu/retnet/) have been found to be associated with RP. Among these 64 RP genes, at least 18 (28%) encode proteins that localize to the cilia in photoreceptors (autosomal recessive RP: *ARL6, BBS1, BBS9, C2ORF71, C8ORF37, CLRN1*, *FAM161A, MAK, TTC8, TULP1, USH2A* and *CEP290*; autosomal dominant RP: *RP1, TOPORS* and *RP1L1*; X-linked RP: *OFD1, RP2, RPGR*) [3, 4]. Cilia are tiny, hair-like microtubule-based cellular organelles that extend outwards from the cell surface. Almost all vertebrate cells have cilia and they serve a variety of sensory functions (in both unicellular and multicellular organisms) [5].

The notion of retinal ciliopathies was first discovered with the observation that patients with X-linked retinitis pigmentosa and Usher syndrome show irregularities in the tails of sperm and in sperm motility [6, 7]. Sperm flagella and photoreceptor cilia share a common axoneme structure. In photoreceptors, cilia are responsible for connecting the outer and inner segments of photoreceptors. There are four ciliary compartments in photoreceptors: the distal cilium, the proximal cilium (known as the connecting cilium), the basal body and the periciliary complex [8, 9]. In addition to its structural function, the photoreceptor cilium plays a critical role in transport. Every minute, an estimated 2,000 opsin molecules are delivered to the outer segments through the cilia [10–12]. Retinal ciliopathies highlight the importance and need for more research on cilia and perhaps a common focus for therapies for ciliopathies.

As of now, specialized genetic counseling and optimizing remaining vision remain essential to RP management. Many promising new therapies are on the horizon and already have clinical trials underway [13, 14]. Thus, there is a great need for studies describing natural disease progression for different types of RP; continued tracking of RP’s progression provides critical data to help create metrics for future clinical trials. Also, such metrics can help with patient counseling for specific types of RP.

FAF images reveal that several RP patients have hyperautofluorescent rings, which are thought to be caused by abnormal lipofuscin accumulation in the perifoveal region of the retinal pigment epithelium (RPE) [15, 16]. The abnormal lipofuscin accumulation could be attributed to defective outer segment regeneration, a precursor of apoptosis in RP. As previously described, SW-AF images have revealed many hyperautofluorescent rings which progressively constrict, correlating with a worsening of visual function over time as measured by pattern electroretinogram (ERG) [17].

Our study evaluated and compared the progression of ciliopathy and non-ciliopathy arRP patients by measuring clinically-relevant parameters including the constriction of hyperautofluorescent rings in FAF images and the progressive shortening of the ellipsoid zone line width obtained by SD-OCT.

## Results

**Fig. 1:**
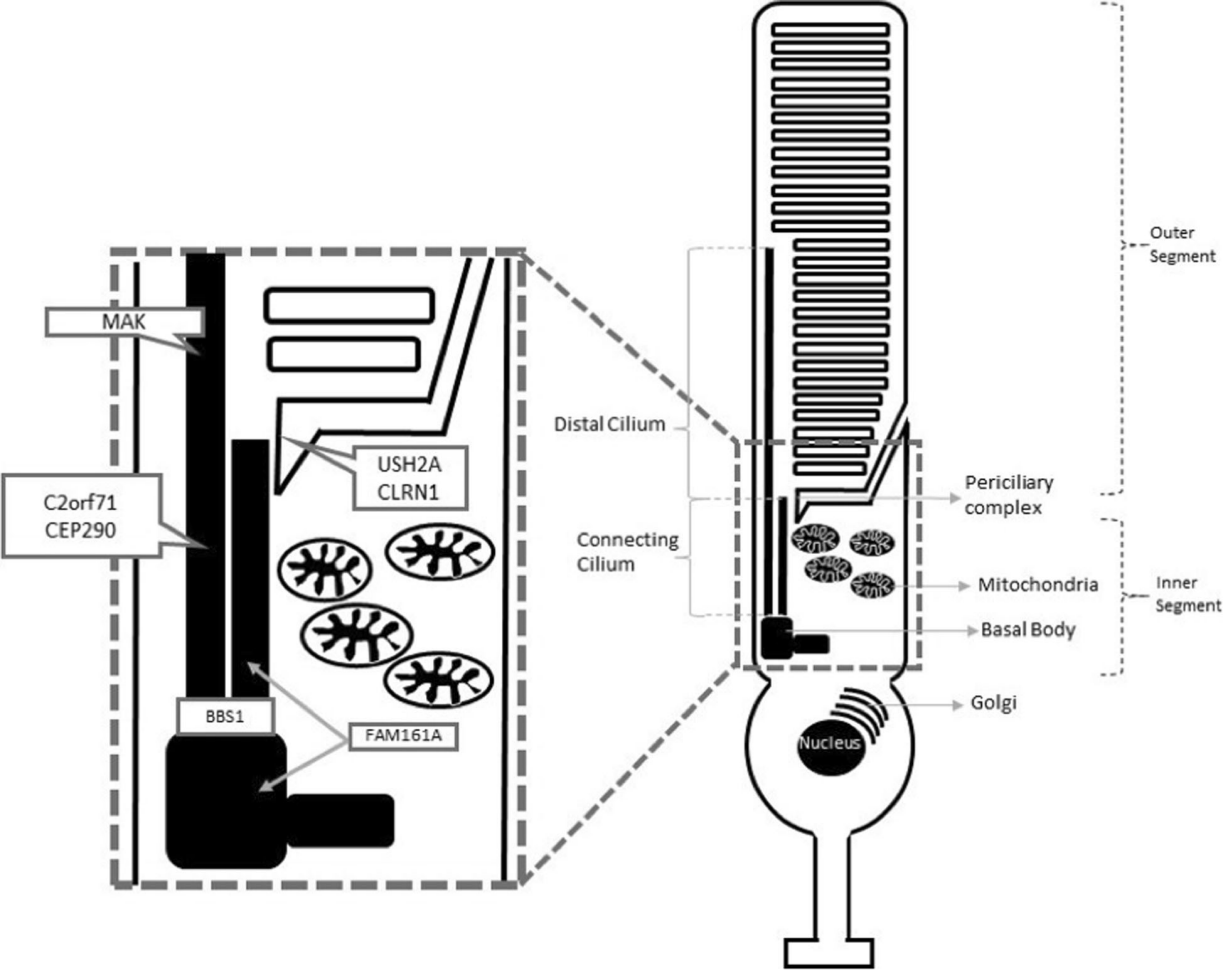
Model of the localization of retinal ciliopathy proteins for each patient included in the study. Four ciliary compartments can be defined in photoreceptors: the distal cilium, the connecting cilium or proximal cilium, the basal body and the periciliary complex. The distal cilium is occupied by MAK. Proteins in the connecting cilium include CEP290and C2orf71. BBS1 is in the basal bodies domain. USH2A and CLRN1 protein is located at the periciliary complex. FAM161A protein was found in the connecting cilium and basal body [3, 4]

After the inclusion and exclusion screening of the 141 index cases with arRP, 18 ciliopathy patients and 15 non-ciliopathy patients were selected. Among the ciliopathy group, mutations were found in 9 *USH2A* patients, 3 *CEP290* patients, 2 *C2ORF71* patients, 1 *FAM161A* patient, 1 *MAK* patient, 1 *BBS1* patient and 1 *CLRN1* patient. A model for the localization of retinal ciliopathy proteins for each of these genes is represented in Fig. 1. Patients’ clinical and genetic details are summarized in Table 1. These 18 patients accounted for a percentage of 12.0% for mutations in ciliary genes in our arRP cohort. The average age of the ciliopathy patients at the first visit was 44 (± 16) years old, and thirteen (72%) patients were male and five (28%) were female. Information regarding age and gender of non-ciliopathy patients are shown in Table 2. The 33 patients were followed for an average of 3.3 (± 2.3 sd) years. The 18 ciliopathy and 15 non-ciliopathy patients were followed for an average of 3.42 (±2.65) and 3.12 (±2.06) years respectively.

**Table 1:**
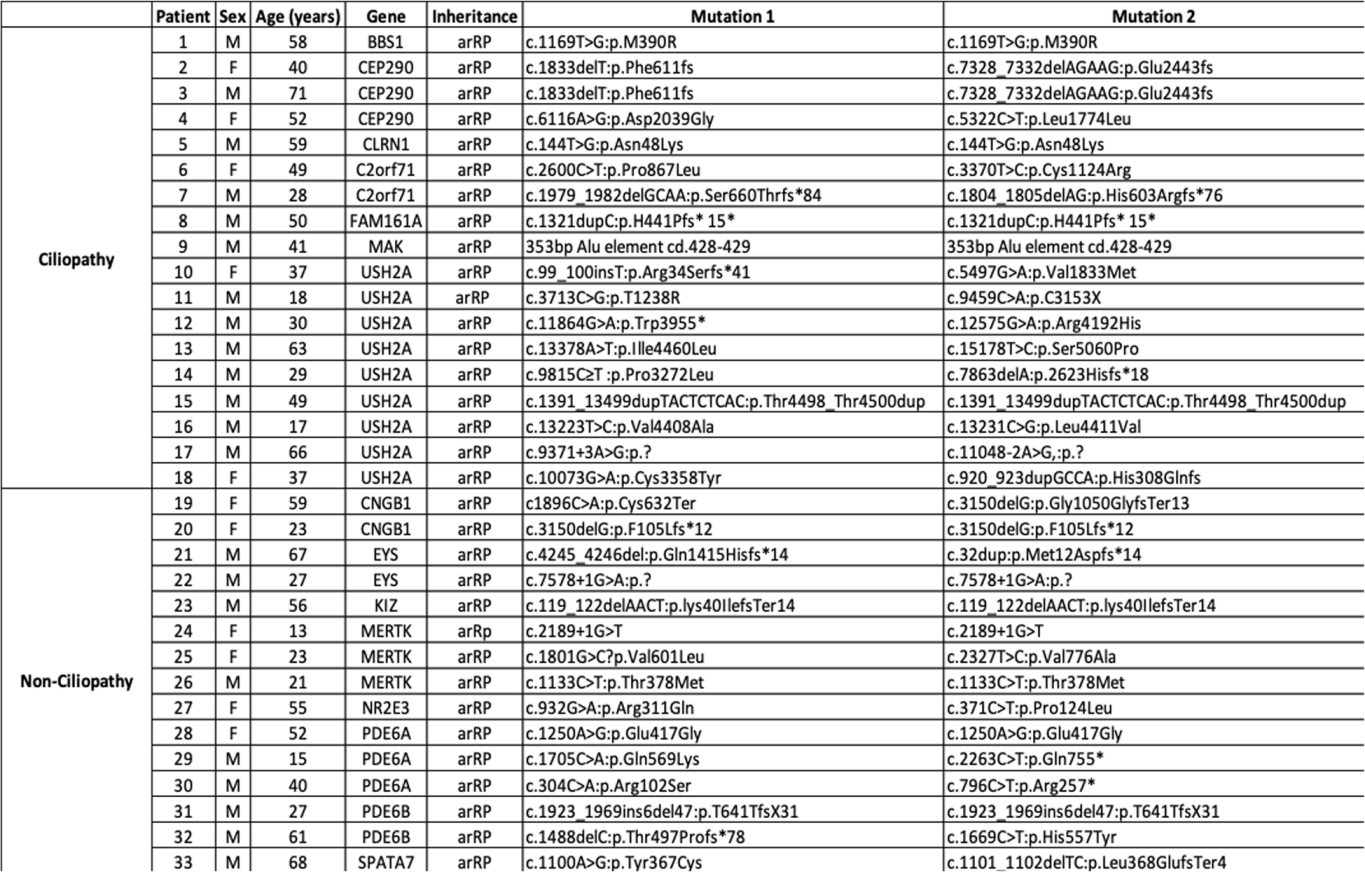
Characteristics of the 33 patients included in the study

**Table 2:** Patients in the ciliopathy group and non-ciliopathy group, with information regarding age and gender

		Sex		Age (years old)					
		Freq. (%)			Quantiles				
	n	M	F	Mean (sd)	Minimum	25th	Median	75th	Maximum
Ciliopathy	18	13 (72)	5 (28)	44 (16)	17	30	45	58	71
Non-ciliopathy	15	9 (60)	6 (40)	40 (20)	13	23	40	59	68

Reliability of the four measurements was analyzed using descriptive statistics (Table 3) and intraclass correlation. The 95th percentile of the absolute value of the difference between the investigators’ measurements was less than 344 μm for horizontal diameter, 329 μm for vertical diameter, 2.2mm^2^ for area and 176 μm for EZ-line width. The intraclass correlation was 0.99 for each of the four measurements, and high intraclass correlation indicates that the measurements were highly reliable.

**Table 3:** Descriptive statistics of the difference between the two graders for structural imaging parameters used to monitor retinitis pigmentosa progression

		Difference	Absolute value of difference between two raters			
	Number of images	Mean (sd)	Mean (sd)	Median (IQR)	95th percentile	ICC
Horizontal diameter (μm)	66	31.3 (167)	131 (106)	113 (49, 181)	344	0.993
Vertical diameter (μm)	66	−9.8 (160)	118 (107)	92.5 (43, 161)	329	0.996
Area (mm^2^)	66	−0.02 (0.9)	0.56 (0.7)	0.31 (0.09, 0.72)	2.2	0.997
EZ line width (μm)	66	−7.9 (96)	77 (57)	66.5 (29, 116)	176	0.997

**Fig. 2:**
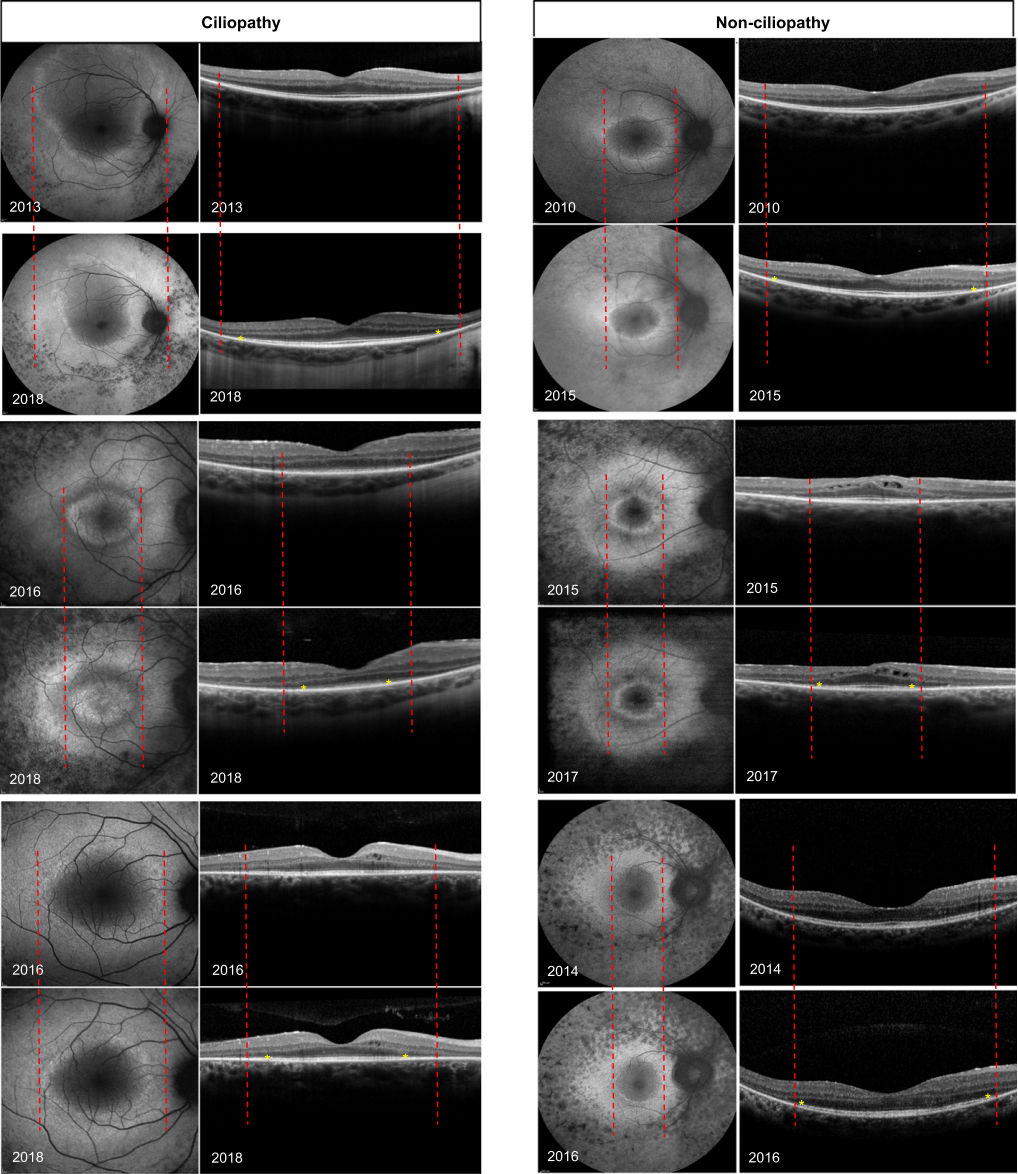
Structural measurements of 6 patients. 3 ciliopathy (right column) and 3 non-ciliopathy (left column) patients. Fundus autofluorescence (FAF) images (left panels) and optical coherence tomography (OCT) images (right panels) monitor progression over time. Dashed lines indicate the initial width of the hyperautofluorescent ring area, and the initial width of the ellipsoid zone line in the OCT images. Yellow asterisks mark the border of the ellipsoid zone lines in the OCT images. Progressive constrictions of the hyperautofluorescent ring and ellipsoid zone line are shown by the constriction of the ring and shortening of the ellipsoid zone line width at 2 different times

Hyperautofluorescent ring dimensions and EZ-line width were obtained from FAF and SD-OCT images as shown in Fig. 2. The structural measurements’ data points were best fit with linear modeling, which provided an estimate of the progression rate of each patient. Progression rate analysis for the right and left eyes of the two groups are shown in Table 4. For the ciliopathy group, the estimated mean shortening of the ellipsoid zone line was 260 μm per year (SD = 162, *p* < 0.001), representing approximately 0.87 degrees of visual field loss per year. The horizontal and vertical diameters decreased at a rate of 351 μm per year (SD = 239, *p* < 0.001) and 348 μm per year (SD = 325, *p* < 0.001), respectively. The ring area decreased at a rate of 2.46mm^2^ per year (SD = 2.64, *p* = 0.001). Represented as a decrease from the mean value of the initial visit, the cohort had a yearly progression rate of 8.0% by EZ-line, 8.1% by horizontal diameter, 8.5% by vertical diameter and 13% by ring area. For the non-ciliopathy group, the estimated mean shortening of the ellipsoid zone line was 84 μm per year (SD = 81, *p* = 0.001), representing approximately 0.3 degrees of visual field loss per year. The horizontal and vertical diameters decreased at a rate of 117 μm per year (SD = 134, *p* = 0.005) and 163 μm per year (SD = 312, *p* = 0.006), respectively. The ring area decreased at a rate of 0.7 mm^2^ per year (SD = 1.63, *p* = 0.11). Represented as a decrease from the mean value of the initial visit, the cohort had a yearly progression rate of 4.5% by EZ-line, 4.0% by horizontal diameter, 7.0% by vertical diameter and 11% by ring area.

**Table 4:** Rates of progression for the four measured parameters (EZ line width, horizontal, vertical diameters, and hyperautofluorescent ring area) for ciliopathy and non-ciliopathy patients

		OD			OS			
		Change over time per year, Mean (sd)	*p*-value^*^	*p*-value **	Change over time per year, Mean (sd)	*p*-value^*^	*p*-value **	Difference in change, Mean (sd)
Horizontal diameter (μm)	Ciliopathy	−351 (239)	< 0.001	0.001	− 317 (208)	< 0.001	0.002	−34 (162)
	Non-ciliopathy	−117 (134)	0.005		−101 (163)	0.03		−15 (56)
Vertical diameter (μm)	Ciliopathy	- 348 (325)	< 0.001	0.11	− 319 (391)	0.003	0.01	−29 (428)
	Non-ciliopathy	−163 (312)	0.06		−55 (38)	< 0.001		−108 (299)
Ring area (mm2)	Ciliopathy	−2.46 (2.64)	0.001	0.03	−2.48 (2.96)	0.002	0.01	0.03 (1.8)
	Non-ciliopathy	−0.71 (1.63)	0.11		−0.47 (0.82)	0.045		− 0.24 (0.85)
EZ line width (μm)	Ciliopathy	−260 (162)	< 0.001	< 0.001	−295 (225)	< 0.001	0.001	35 (156)
	Non-ciliopathy	−84 (81)	0.001		−88 (69)	< 0.001		3.6 (62)

* One sample t test for testing whether mean change over time is different from 0

** Two sample t test for comparing mean change over time between Ciliopathy vs. Non-ciliopathy

The correlation between the four parameters measured at the initial visit for the 33 patients was calculated (Table 5). The highest correlation coefficients observed were between the vertical diameter and area (*r* = 0.95) and between the horizontal diameter and vertical diameter (*r* = 0.94). The lowest correlation coefficient observed was between the area and the EZ line width (*r* = 0.82).

**Table 5:** Correlations between the four different parameters, at the initial visit for the 33 patients, were calculated: EZ line width, horizontal, vertical diameters, and hyperautofluorescent ring area of OD

Correlation between parameters		Correlation coefficient (r)	*P*-value^*^
Horizontal diameter	Vertical diameter	0.94	<0.001
Horizontal diameter	Area	0.84	<0.001
Horizontal diameter	EZ line width	0.93	<0.001
Vertical diameter	Area	0.95	<0.001
Vertical diameter	EZ line width	0.91	<0.001
Area	EZ line width	0.82	<0.001

Asymmetry between left and right eye disease severity was seen at baseline [18], and asymmetric progression of the four parameters between the two eyes was assessed during follow-up. The difference in average progression for the ciliopathy patients was 34 μm for the horizontal diameter, 29 μm for the vertical diameter, 0.02mm^2^ for the ring area, and 36 μm for the EZ line length. The non-ciliopathy patients presented a difference of 16 μm for the horizontal diameter, 108 μm for the vertical diameter, 0.24 mm^2^ for the ring area, and 3 μm for the EZ line length.

## Discussion

Ciliary gene mutations can result in an extensive range of clinical features that manifest in the central nervous system, eye, heart, liver, gonads, kidney, adipose tissue and bones. Based on multiple clinical features that involve these diverse organs, various syndromes have been defined, such as Bardet-Biedl syndrome, Joubert syndrome, and McKusick-Kaufman syndrome [19, 20]. Retinal dystrophy can present as one of the clinical features of these syndromes, but it is more often an isolated disease that presents without additional features.

Hyperautofluorescent ring constriction is related to visual loss in RP patients, and it could be used as a prognostic for the retention of central vision [15]. Previous studies have shown that the presence and rate of ring constriction are likely to be genotype dependent [21]. In this study, we compared the disease progression in autosomal recessive RP patients with and without ciliary gene mutations by measuring four structural parameters as markers of degeneration: EZ line width from SD-OCT images, horizontal diameter, vertical diameter and hyperautofluorescent ring area from FAF images. We report that in the ciliopathy arRP patients, the EZ line width decreases at a rate of 259 μm (0.8 degrees) per year, 32.5% faster than the non-ciliopathy group. The hyperautofluorescent ring also constricts over time, with the horizontal and vertical diameters decreasing at a rate of 351 μm and 347 per year, respectively. This represents a progression rate that is 33 and 47% faster for the horizontal and vertical diameter, respectively. The ring area decreases at a rate of 2.46mm^2^ per year, which is 28% faster than the non-ciliopathy patients. Out of the four parameters, our results demonstrate that arRP patients with the mutation in the ciliary-genes progress faster than arRP patients with non-ciliary-genes.

A 2015 study analyzed 71 RP patients, 48 (67.6%) with arRP but only 6 (8%) with ciliary gene mutations, and the EZ line width was reported to decrease at an average rate of 130 μm (0.45 degree) per year, while the horizontal and vertical diameter decreased at a rate of 147 μm per year and 121 μm per year [22].A more recent study in 2017 analyzed 81 RP patients of which 41 (50.6%) had arRP and only 2 (2.5%) had ciliary gene mutations. In this study, the EZ line width was reported to decrease at a rate of 140 μm (0.45 degree) per year, while the horizontal and vertical diameters decreased by 149 μm and 120 μm per year [23]. Traditionally, X-linked retinitis pigmentosa (XLRP) is known to progress faster than arRP and adRP, with adRP demonstrating the slowest progression [2, 24]. Mutations in the retinitis pigmentosa GTPase regulator (*RPGR*) gene are associated with RP that is often transmitted in an X-linked manner [25].*RPGR* mutations account for the disease in over 70% of XLRP patients [26] and the constitutive variant of *RPGR* is believed to be expressed in a wide variety of tissues including the connecting cilia of rods and cones, the transitional zone of cilia of the respiratory epithelium, the epithelial lining of human bronchial and sinus tissues, and the human fetal cochlea [27].A more homogenous cohort was analyzed by a study that compared progressive loss of the EZ line in adRP and XLRP patients [28]. The study included 26 XLRP patients, of which 25 had an *RPGR* gene mutation and 1 had no available genetic testing results. The study reported a faster rate of progression in XLRP with a EZ line width of 1 degree per year. This result is very similar to our EZ line width in the arRP ciliopathy group (0.87 degree/year), which was expected since *RPGR* is a ciliopathy gene.

We believe that the more severe loss of EZ line width and SW-AF ring constriction found in RP ciliopathy patients compared to non-ciliopathy patients is related to the important function of cilia in photoreceptors. The outer segments of photoreceptors are unable to synthesize essential proteins and lipids, and all phototransduction proteins and disc membrane lipids must be synthesized in the inner segment and then transported to the outer segment through the cilia system. With the constant turnover of rod outer segments, delivering cargo to the outer segments is essential for maintenance of the outer segments [10–12].

In patients with two recessive mutations that create a diseased phenotype, gene supplementation therapy uses a viral vector to introduce a wild-type allele that would allow the cells to have sufficient expression of the desired normal gene product [29]. In gene therapy clinical trials, one eye typically serves as a control while the contralateral eye receives treatment. Assuming that disease progression is symmetric between the eyes, this provides the opportunity to compare the treated eye to a near-ideal control. In our study, we found that the right and left eyes have symmetrical progression rates, suggesting minimal asymmetry.

As a limitation to this study, only patients with high-quality FAF and SD-OCT scans were analyzed in order to produce an accurate analysis. This is a problem for patients with advanced RP as these patients lack good fixation due to poor vision. Thus, patients with advanced RP were excluded in order to acquire high-quality scans for analysis. This limits the possibility of studying changes in the retina in patients with advanced RP. In addition, among of our cohort of 18 ciliopathy patients, 9 had *USH2A* mutations, and this can cause an impressive rate of progression. The *USH2A* gene is the most prevalent of all arRP genes, responsible for 9.5–13% of the cases [30].

## Conclusion

In conclusion, our study was able to quantify and compare the loss of EZ line width and SW-AF ring constriction progression over time for patients with ciliopathy and non-ciliopathy arRP mutations. These results may serve as a basis for modeling RP disease progression, and they could be useful as clinical trial endpoints for studies seeking to promote cone and rod survival in RP patients.

## Methods

### Subjects

The study was conducted in accordance with the principles of the Declaration of Helsinki. All study procedures were defined, and patient consent was obtained as specified by the protocol #AAAR0284 approved by the Institutional Review Board at Columbia University Medical Center. None of the data presented in this study, including images and genetic testing results, are identifiable to individual patients. Longitudinal follow-up imaging of 141 patients with arRP was analyzed. The patients were divided into two groups according to gene mutation: ciliary genes and non-ciliary genes. Patients were diagnosed with RP by an inherited retinal disease specialist (SHT) based on their clinical history, symptoms, past family history, fundus findings, and full-field electroretinography (ffERG). The diagnosis was supported by clinical imaging and/or genetic testing. In addition, each patient was screened for a history of 2 visits in our office at least 12 months apart consisting of a complete ophthalmic examination by a retinal physician (SHT). The patients excluded were those who presented with unilateral RP, no visible EZ line, no visible hyperautofluorescent ring or poor image quality. Because our clinic is an international referral center for RP, after the initial diagnosis was made for a large number of patients using ffERG and clinical imaging and/or genetic testing results, care was transferred back to the primary provider, and patients did not return for a second visit.

### Fundus autofluorescence and spectral-domain optical coherence tomography

The images were acquired at each visit after pupil dilation with phenylephrine hydrochloride (2.5%) and tropicamide (1%). The FAF (488 nm excitation) and SD-OCT imaging were acquired with the Spectralis HRA + OCT (Heidelberg Engineering, Heidelberg, Germany). FAF imaging was acquired with a 30-degree field of view and the 55-degree field of view was used in cases where large rings could not be fully captured with the 30-degree field of view.

Measurements were done on the SD-OCT and FAF images documented at every patient visit by using a built-in measurement tool in the Spectralis HRA + OCT software. EZ line length, horizontal diameter, vertical diameter, and area of hyperautofluorescent ring were measured by two ophthalmologists (V.K.L.T and M.B.A). The horizontal diameter was defined as the line positioned at the axis formed by the distance between the center of the optic disc and foveal center. The vertical diameter was positioned perpendicularly to the horizontal diameter. The delineable edge of the hyperautofluorescent ring was used as the border to measure the area of the ring (Fig. 1). On the SD-OCT, the nasal and temporal edges of the EZ line were defined as the locations where the EZ line met the RPE. The width of the EZ line was defined as the distance between these two locations.

### Statistical analysis

Statistical analyses were performed using Stata 12.1 (StataCorp, College Station, TX, USA) software. Analyses were done separately for right eye and left eye. Where results are similar, we present results for the right eye. The reliability of test-retest measurements was assessed using summary/descriptive statistics and intraclass correlation coefficients (ICC). Given the high ICC coefficients of the two investigators’ measurements, an average value was obtained from the two measured values and used for further data analysis. The simple Pearson correlation coefficient was calculated between different structural measurements from the initial visit. Change over time was calculated by taking the value of an ophthalmologic outcome at follow-up minus the value at baseline and then dividing by the time of follow-up. To examine whether there was asymmetry between right eye vs. left eye, we took the change over time in the right eye and subtracted the change over time in the left eye. Progression, change over time, was examined for right and left eyes separately. A Student’s t-test was performed to test for a difference of the progression rates from zero, within a specific group, ciliopathy or non-ciliopathy. To compare mean change over time between groups, two sample t tests were used.

## Data Availability

The datasets generated and/or analyzed during the current study are not publicly available due to privacy but are available from the corresponding author on reasonable request.

## Abbreviations

arRP: Autosomal recessive Retinitis Pigmentosa
ERG: Electroretinogram
FAF: Fundus autofluorescence
ffERG: Full-field electroretinography
ICC: Intraclass correlation coefficients
RP: Retinitis Pigmentosa
RPE: Retinal pigment epithelium
*RPGR*: Retinitis pigmentosa GTPase regulator
SW-AF: Short-wavelength autofluorescence
XLRP: X-linked retinitis pigmentosa
